# Exposures during the prepuberty period and future offspring’s health: evidence from human cohort studies^†^

**DOI:** 10.1093/biolre/ioab158

**Published:** 2021-08-20

**Authors:** Cecilie Svanes, Randi J Bertelsen, Simone Accordini, John W Holloway, Pétur Júlíusson, Eistine Boateng, Susanne Krauss-Etchmann, Vivi Schlünssen, Francisco Gómez-Real, Svein Magne Skulstad

**Affiliations:** Department of Global Public Health and Primary Care, Centre for International Health, University of Bergen, Bergen, Norway; Department of Occupational Medicine, Haukeland University Hospital, Bergen, Norway; Department of Clinical Science, University of Bergen, Bergen, Norway; Oral Health Centre of Expertise Western Norway, Bergen, Norway; Unit of Epidemiology and Medical Statistics, Department of Diagnostics and Public Health, University of Verona, Verona, Italy; NIHR Southampton Biomedical Research Centre, University Hospital Southampton, UK; Human Development and Health, Faculty of Medicine, University of Southampton, Southampton, UK; Department of Clinical Science, University of Bergen, Bergen, Norway; Department of Health Register Research and Development, National Institute of Public Health, Bergen, Norway; Early Life Origins of Chronic Lung Disease, Research Center Borstel, Leibniz Lung Center, German Center for Lung Research (DZL), Borstel, Germany; Early Life Origins of Chronic Lung Disease, Research Center Borstel, Leibniz Lung Center, German Center for Lung Research (DZL), Borstel, Germany; Institute of Experimental Medicine, Christian-Albrechts-Universität zu Kiel, Kiel, Germany; Department of Public Health—Work, Environment and Health, Danish Ramazzini Centre, Aarhus University, Denmark; National Research Centre for the Working Environment, Copenhagen, Denmark; Department of Clinical Science, University of Bergen, Bergen, Norway; Department of Gynaecology and Obstetrics, Haukeland University Hospital, Bergen, Norway; Department of Occupational Medicine, Haukeland University Hospital, Bergen, Norway

**Keywords:** adolescence, allergies, anthropometry, asthma, father’s overweight, father’s smoking, nongenetic heredity, obesity, prepuberty, puberty, RHINESSA, sex-specific

## Abstract

Emerging evidence suggests that exposures in prepuberty, particularly in fathers-to-be, may impact the phenotype of future offspring. Analyses of the RHINESSA cohort find that offspring of father’s exposed to tobacco smoking or overweight that started in prepuberty demonstrate poorer respiratory health in terms of more asthma and lower lung function. A role of prepuberty onset smoking for offspring fat mass is suggested in the RHINESSA and ALSPAC cohorts, and historic studies suggest that ancestral nutrition during prepuberty plays a role for grand-offspring’s health and morbidity. Support for causal relationships between ancestral exposures and (grand-)offspring’s health in humans has been enhanced by advancements in statistical analyses that optimize the gain while accounting for the many complexities and deficiencies in human multigeneration data. The biological mechanisms underlying such observations have been explored in experimental models. A role of sperm small RNA in the transmission of paternal exposures to offspring phenotypes has been established, and chemical exposures and overweight have been shown to influence epigenetic programming in germ cells. For example, exposure of adolescent male mice to smoking led to differences in offspring weight and alterations in small RNAs in the spermatozoa of the exposed fathers. It is plausible that male prepuberty may be a time window of particular susceptibility, given the extensive epigenetic reprogramming taking place in the spermatocyte precursors at this age. In conclusion, epidemiological studies in humans, mechanistic research, and biological plausibility, all support the notion that exposures in the prepuberty of males may influence the phenotype of future offspring.

## Teaser

Emerging research from human multigeneration cohorts, historic studies, and mechanistic research indicates that prepuberty is an important window of susceptibility with regard to health and disease of future offspring.

## Introduction and aims

The vast majority of public health strategies are aimed at improving health or reducing disease within the life span and usually in the near future. In recent decades, it has become generally acknowledged that early life conditions, in particular the intrauterine environment, may have lifelong impact [1–7]. This is reflected in “First 1000 Days” programs around the world targeting the mother and child. Current multigeneration research has provided evidence that exposures during pregnancy may also impact grand offspring [8–14]. Thus, the focus on the pregnant woman might in some cases give triple returns—for the unborn child, for the mother beyond pregnancy, and, possibly, for the grand offspring whose germ cells are present already in the fetus in utero. Prepuberty is a time window with major changes preparing the body for reproductive life. Could prepuberty be another susceptibility window, in which interventions could benefit both the target person and future offspring?

There are historic studies that support the notion that prepuberty may be an important time window with regard to health in future generations. Bygren et al. [15] linked ancestral food availability during different age windows to mortality data for grand-offspring and found that paternal grandfather’s food shortage during prepuberty was associated with grandchildren’s higher longevity. Analysis of one out of two other birth cohorts from Överkalix confirmed this finding [16, 17], as did analyses of much later birth cohorts from Uppsala [18]. A study of mental health in descendants of persons who survived the German famine winter 1916–1917 found associations of grandparental prepuberty exposure to famine with better mental health outcomes in grand-offspring, following sex-specific patterns within the male and female lines [19]. These studies generally suggest positive health outcomes related to paternal grandfather (and, to some extent, grandmother) living through food shortage in prepuberty.

The paradigm of early life origins of health and diseases, later named the Developmental Origins of Health and Disease (DOHaD), has been critical to the understanding of development of chronic diseases [1–7]. Traditionally, the main focus has been on mother and child. A new paradigm on the role of the father, the paternal origins of health and disease (POHaD), has been introduced the last years [20–24], implying biologically shared responsibility of both sexes for future generations’ health.

Prepuberty may be a susceptibility window relevant for both boys and girls and their future offspring [20]. Addressing this specific time window is hampered by several challenges: (i) birth cohorts often have good data on mothers, little on fathers, and limited data back in time from the prepuberty of both parents; (ii) animal models are difficult, for example, puberty in mice lasts only few days; and (iii) mature sperm can be studied in humans, whereas eggs are less feasible to obtain, from both sexes it is practically impossible to study germline precursor cells from human prepuberty. Despite these challenges, there are valuable scientific advancements from human and animal studies, epigenetic research, and methodological work that underpin the notion that parental prepuberty is an important time window with regard to the health and disease of future offspring.

**Figure 1:**
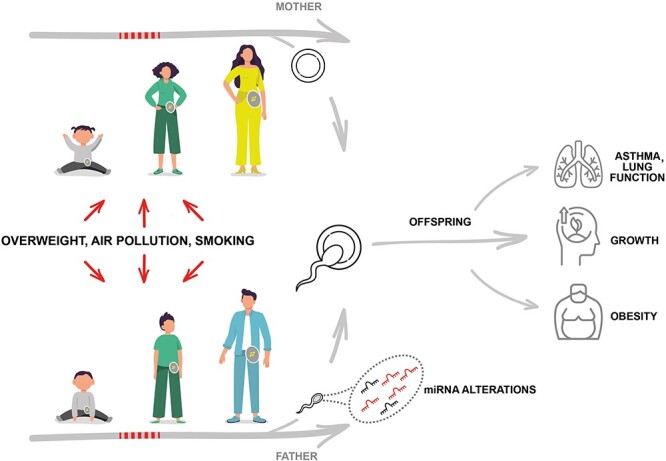
Exposures such as overweight and smoking may influence individuals as well as their germ cells, during any life stage from early life and through reproductive life. The germ cells may transfer a “fingerprint” of exposure effects that may influence offspring phenotype. Prepuberty may be a time window of particular susceptibility.

The aim of this paper is to review the literature on exposures during the prepuberty period as related to future offspring’s health in human studies (Figure 1), discuss potential mechanisms and the solidity of this evidence basis, and attempt to identify needs for further research. Specifically, we will address puberty, human cohort studies with specific emphasis on the RHINESSA studies, mechanistic work and animal studies, discussion, and conclusions and public health implications.

## Puberty

Extensive alterations in bodily processes culminate in puberty, when boys and girls enter fertile age. Puberty is a period of physical and psychological maturation, associated with long-term health outcomes [25–30]. Growth and development of children are prone to secular trends and respond to a complex mix of societal and environmental factors. Adult stature was reported to increase in European countries in the 20th century [31] and still appears to be increasing in Nordic populations [32]. The age of menarche was reported to decline in the 19th century and the first half of the 20th century in Western countries [33], a trend that was attributed to better general health with improved nutrition and hygiene [34]. More recent data indicate that the onset of puberty is still occurring in younger ages [35]. A recent Norwegian study has documented ongoing reduction in the age of menarche even from 2003–2006 to 2016 [36]. Trends toward more rapid maturation have also been reported in boys, albeit the magnitude of these changes is less pronounced than in girls [37]. Several factors have been proposed to explain this trend toward earlier puberty development in recent times. Evidence suggests that weight status affects the timing of puberty [38–40]. Adoption, immigration, deprivation, and abuse have been reported to be associated with early pubertal development, possibly secondary to psychosocial stress [41, 42]. Environmental factors such as exposure to endocrine-disrupting chemicals (EDCs) are also believed to play a role in the secular change [43].

The timing of pubertal onset appears to have profound impact on future health. Early menarche has been related to increased risk for adult asthma and low lung function [28], cardiovascular disease, different types of cancers, obesity, and all-cause mortality [44]. Early onset of puberty in boys has also been related to increased risk for cardiovascular events, type 2 diabetes, and hypertension [44]. In both genders, early pubertal development has been related to negative psychosocial and behavioral outcomes [45].

### Germ cell development in humans

The first cells of the germ lineage are called primordial germ cells (PGCs). PGCs occur in the peri-implantation human embryo around the time of gastrulation [46–48], very early in the embryonal development when the embryo has begun differentiation to establish distinct cell lineages. Human PGCs originate during amnion specification and also from the posterior end of the nascent primitive streak [46, 49]. Following specification, PGCs migrate into the genital ridges at 4–5 weeks postfertilization [48]. At 6 weeks postfertilization, the genital ridges differentiate into either the male or female gonads, with sex-determining region on the Y chromosome being essential for testicular development in males [50–52]. Kota et al. describe key epigenetic transitions in male and female germ cell development [53].

One of the earliest morphological changes in *the male gonad* at 6 weeks is the formation of nascent "cord-like" structures comprising PGCs and Sertoli-lineage cells surrounded by fetal Leydig and interstitial cells. In humans, this basic niche structure persists through the fetal and postnatal stages; the formation of an organized seminiferous tubule does not occur until the pubertal stages in humans [54, 55]. Within the developing fetal testicular niche, male germline cells undergo major developmental changes [56–59]. Notably, there is transitions from pluripotent-like PGCs migrating to and into the developing gonad, to pluripotent-like and mitotically active PGCs in the gonad (called fetal germ cells or gonocytes), followed by transition to "mitotically arrested" germ cells that repress the pluripotency-like program from weeks 14 to 18 [58]. The mitotically arrested germ cells that arise during weeks 14–18 are nearly identical with the postnatal spermatogonial stem cells (SSCs). In the adult testis, five distinct spermatogonial states with increasing level of differentiation are defined, with state 0 being the most naive and undifferentiated state [54, 60, 61]. This state is the predominant SSC state present in the infant testis, and the mitotically arrested prenatal germ cells transcriptionally resemble state 0 postnatal spermatogonia [62]. The testis niche plays an important role in guiding the survival and differentiation of the male germline. The development of the functional adult testis and its organized tubule-like structure is completed at puberty, during which the final specification and maturation of all somatic niche cells takes place. Single-cell RNA (scRNA)-sequencing to study human testis development during puberty has revealed a common progenitor for Leydig and myoid cells that exists before puberty in humans, which is analogous to the somatic progenitor observed in fetal mice [54].

After sex determination, *female PGCs* arrive at the developing ovaries and proliferate with an incomplete cytoplasmic mitosis to form oogonia cysts and subsequently initiate meiosis to become meiocytes [63]. The developmental trajectories of embryonic female germ cells are described both in humans and mice [57, 58, 64, 65]. The mitosis to meiosis transition is accompanied by drastic changes in transcriptomes and represents a crucial event during early female germ cell development. Mitotic female germ cells are relatively homogeneous and exhibit minor developmental stage-specific expression patterns [57]. After entering meiosis, the transcriptome in meiotic oocytes is sharply different from that in mitotic oogonia, and some of the germline marker genes exhibit obvious developmental stage-specific expression pattern. Primordial follicle assembly in mammals occurs at the embryonic stage in human ovaries, which constitutes the ovarian reserve responsible for the reproductive lifespan. There are two waves of primordial follicle assembly in humans, and the first wave occurs following the retinoic acid responsive stage, while the second wave takes place at the late meiotic prophase [66]. The stage-specific gene expression patterns revealed by single-cell RNA sequencing reflect the physiological status of oocyte growth and maturation [67].

### Epigenetic reprogramming during germ cell development

While one may previously have considered germ cells mainly a source of DNA to be transmitted to offspring, we today know that epigenetic material and processes are fundamental and also transmitted to offspring. Known molecular mechanisms that contribute to epigenetic inheritance are DNA methylation, histone modifications, and noncoding RNAs (ncRNA) [68]. Studies of rodents suggest that transgenerational epigenetic inheritance through gametes can be modulated by environmental factors. Modification and redistribution of chromatin proteins in gametes are perhaps the major routes for transmitting epigenetic information from parents to the offspring [69].

The first reprogramming of the epigenome occurs during early embryonic development from the zygote stage to the formation of layers, and the second one occurs during the somatic-to-germline transition [53]. Human PGCs show transcription patterns involving the simultaneous expression of both pluripotency genes and germline-specific genes. Approximately 10–11 weeks after gestation, the PGCs are nearly devoid of any DNA methylation. The median methylation levels in male and female PGCs are reported to be only 7.8% and 6.0%, respectively, indicating a global demethylation of the genomes of the PGCs [57]. The evolutionarily younger and more active transposable elements have been found to maintain higher levels of residual methylation than the older and less active ones, meaning that the evolutionarily younger transposable elements are transcribed more actively than the evolutionarily older ones.

Notably, there are two main differences between females and males when it comes to the epigenetic reprogramming: Global remethylation in female PGCs starts at 11 weeks of gestation, which is much earlier than in male PGCs [57]. Further, in males, a third event occurs during spermatogenesis, starting at puberty. In practical terms, this implies different susceptibility windows between the sexes, and it also signals that young men may be more vulnerable when it comes to environmental influences in puberty than young women.

## Human cohort studies with specific emphasis on the RHINESSA studies

Only few human cohorts have data on parental prepuberty exposures for well-characterized offspring. Below is a description of the RHINESSA study, which was designed to investigate preconception exposures.

### The RHINESSA population-based multigeneration study

The RHINESSA study (Respiratory Health in Northern Europe, Spain and Australia, www.rhinessa.net) investigates the influence of exposures before conception and in previous generations on health and disease, in order to identify preconception susceptibility windows and explore mechanisms for exposure effects across generations. RHINESSA is a multigeneration multicenter population-based study that focuses mainly on asthma, allergies, and respiratory health but has data and methodology that are valuable for a range of health outcomes.

As the human reproductive cycle spans decades, investigating exposure effects from before conception and across generations in human cohorts represents a great challenge. The RHINESSA study is designed to address this by investigating the offspring of persons who have been extensively characterized for 20 years, spanning their reproductive period. The study allows for unbiased identification of family members plus additional data on exposures and outcomes from excellent national registries in Northern European study centers, and reasonable generalizability of study results by including study centers from Spain, Australia, and Northern Europe.

**Figure 2:**
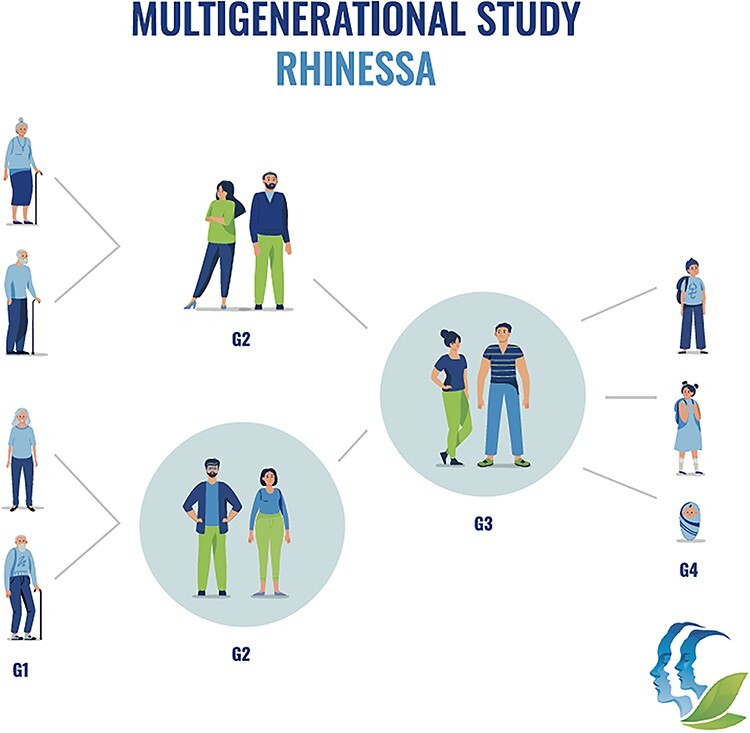
The study design of RHINESSA (Respiratory Health In Northern Europe, Spain and Australia). In 10 study centers, the RHINESSA study has investigated the G3 offspring (*N* = 10 133/1607, questionnaire/clinical) of the G2 participants of the ECRHS and RHINE studies. ECRHS and RHINE have followed G2 participants over 20 years of childbearing years. In one study center, the Bergen RHINESSA study centre, also G1 and G4 has been investigated clinically, as well as the G2 other parent that was not included in the ECRHS/RHINE studies. Study participants of all generations have provided information about themselves, their parents, and potential offspring.

RHINESSA (Figure 2) includes 10 133 persons from 10 study centers in Bergen (Norway), Gothemburg, Umeå and Uppsala (Sweden), Aarhus (Denmark), Reykjavik (Iceland), Tartu (Estonia), Melbourne (Australia), and Albacete and Huelva (Spain). Their parents are participants in the large longitudinal population-based studies of respiratory health in adults, the European Community Respiratory Health Survey (ECRHS, www.ecrhs.org) [70–73] established in the early 1990s, and the linked study Respiratory Health in Northern Europe (RHINE, www.rhine.nu) [74, 75]. For a range of exposures and lifestyle factors, the ECRHS and RHINE cohorts have data with high time resolution before and after conception and birth of the RHINESSA offspring.

### Ethics statement

Ethical permissions were obtained from the local ethics committee in each of the participating centres, for each study wave/group (see www.rhinessa.net). All study participants (or parents/guardian for children and adolescents) provided written informed consent prior to participation, when relevant, including permission to extract information about themselves and family members from national registers.

### Father’s smoking and overweight in prepuberty, and offspring’s asthma and lung function

A first explorative analysis [12] questioned whether father’s smoking *before* conception was associated with asthma in future offspring, and, if confirmed, whether age of starting smoking, years and amount smoked, or interval from quitting until conception of the offspring were more important. This analysis included data for >24 000 offspring of RHINE study participants and thus enable separation of different characteristics of parental smoking. Father’s smoking starting before the age of 15 was by far the feature of parental smoking that was most consistently and strongly associated with offspring’s asthma. More than 10 years of preconception smoking was also associated with offspring’s asthma, while time of quitting or numbers of cigarettes smoked daily were not. No effect was found for mothers’ smoking before conception/in prepuberty, but her smoking around the time of pregnancy showed a similar association with offspring’s asthma as found in other studies. This is reassuring with regard to the novel findings on father’s exposure, given the similar definitions of exposures, outcomes, and covariates as well as similar analytical models of the maternal and paternal lines. Offspring’s asthma was also associated with paternal grandmother’s smoking in this analysis of >24 000 offspring. Intriguingly, paternal grandmother’s smoking significantly modified the associations with father’s smoking; if she had smoked, there was no association of father’s prepuberty smoking with offspring’s asthma, but effects of father’s later onset preconception smoking remained.

The finding of higher asthma risk in offspring if the father had started smoking before age 15 was replicated by Accordini et al. in a multigeneration analysis of the ECRHS cohort [13]. Statistical mediation models were used to account for the complexity in the multicenter multigeneration data, including simulation analyses showing that the impact of unmeasured confounding on the estimates was limited. State-of-the-art statistical methods for causal inference from observational data were applied in a subsequent analysis of the RHINESSA/ECRHS cohorts, finding that father’s smoking before age 15 years might also have a negative impact on lung function in offspring [14]. Impact on both FEV_1_ and FVC suggests detrimental causal effects on lung growth as well as on airways obstruction. Lung function is an important measure of general as well as respiratory health that predicts morbidity and mortality.

Further support for early male puberty as an important susceptibility window was obtained in a study of parents’ overweight starting by childhood, puberty, or young adulthood. Johannessen, Lønnebotn, Calciano et al. found that father’s onset of overweight before voice break (but after age 8 years) might be related to asthma in future offspring [76]. The statistical methodology used suggests that this effect could be causal. Lønnebotn et al. showed that father’s onset of overweight before voice break also appeared to be causally related with lower lung function in offspring [77].

Analyses of the RHINESSA/RHINE/ECRHS cohorts thus suggest, consistently, that both fathers’ early smoking debut and fathers’ overweight in prepuberty may be of key importance for asthma and lung function (Figure 1) [12–14, 76, 77].

### Parental air pollution in childhood/adolescence, and offspring’s asthma and hayfever

Kuiper et al. [78] analyzed parental air pollution in childhood/adolescence as related to offspring’s asthma and hayfever, using RHINESSA data from study centers with available parental exposure data at the time of study. Data on various air pollutants in parents from 1975 onwards were generated by geocoding of parental individual residential addresses obtained from national registries [78]. Parental exposure before age 18 years was analyzed, as the complex data structure did not allow specification of the prepuberty time window. The analysis found that mother’s PM10 exposure in childhood/adolescence was associated with a doubled risk of asthma in her future offspring and that father’s ozone exposure in childhood/adolescence was associated with increased risk for hayfever in offspring’s [78].

### Parents’ early onset smoking and offspring’s overweight/fat mass

Based on data from the RHINESSA study, Knudsen and colleagues found an association between paternal onset of smoking at age <15 with excessive fat mass in their future sons [79]. Paternal and maternal smoking before conception were associated with higher BMI in offspring, but these effects were mediated through parents' pack years of smoking, thus not specifically pointing to the parental prepuberty period. An analysis of the large HUNT study could not identify an association between onset of smoking <11 years with BMI of the offspring; this study did not address fat mass [80]. Analysis of the ALSPAC study showed that if the father had started smoking before age 11, his sons but not daughters had a higher mean BMI at age 9 [16]. Data from follow-up of the offspring until age 17 years found that father’s early onset smoking was associated with fat mass and not lean mass [81]. Subsequent analysis of both the maternal and the paternal lines when offspring reached 24 years confirmed excessive fat mass in offspring if parents started smoking in prepuberty [82]. However, the susceptible age period differed between the paternal and maternal line; for fathers, the susceptibility window was before age 11, but for mothers, 11–15 years. Mother’s early onset smoking was more strongly related to excess body fat in daughters [82]. Consistent with the study of Knudsen et al., father’s early onset smoking was associated with higher fat mass in sons.

Thus, a role for fathers’ early onset smoking for sons’ fat mass is remarkably consistent in analyses of RHINESSA and ALSPAC, even if definitions of father’s overweight differed in these studies. In addition, analyses of ALSPAC demonstrate associations of mother’s smoking onset at age 11–14 with daughters’ fat mass—this is not yet replicated. The results are inconsistent regarding offspring’s BMI; a possible explanation could be that BMI is a more complex outcome than fat mass because the weights lean mass, bone mass, and water are included together with the fat mass.

### Parental prepuberty nutrition and overweight, and offspring’s growth

In a study of food supplementation in children from Guatemala [83], children in one village were given a more nutritious supplement than the children of the other village. When these children came into childbearing age, the offspring of mothers having received the more nutritious food supplement in childhood had higher birth weight, head circumference, and height than the children of the mothers who had received the less nutritious supplement. No such associations were found for father’s childhood nutritional supplement.

In the RHINESSA study populations, overweight rather than undernutrition is the common problem of malnutrition. Analyses of RHINESSA found that maternal overweight at age 8 years and/or at menarche was associated with increased childhood overweight in offspring [76]. With regard to the male line, father’s overweight in childhood was significantly associated with childhood overweight in his future offspring [76].

Thus, better nutrition and normal weight (as opposed to overweight) in parents’ childhood were related to better health outcomes in their future offspring.

## Mechanistic work and animal studies

### Multigenerational animal studies on prepuberty exposure

The human studies described above are mostly based on observational data. Randomization of exposures during parental puberty is ethically impossible, and waiting to see the consequences of such exposure on future offspring would usually not be feasible. Thus, there is a need for animal models as they allow to study exposures in a highly controlled setting and relate these to outcomes in offspring within a relatively short time frame. In addition, tissues including ovary and testes are readily accessible to mechanistic studies.

Data from animal models have clearly demonstrated that epigenetic processes can account for the transmission of the effects of parental exposure (even transient exposure) to their offspring and subsequent generations. [20, 68, 84–87] However, there are only few studies specifically addressing the time window around puberty. The equivalent of puberty is hard to pin down in flies and remains a challenge in mice. Recent work in *D. melanogaster* showed that preconceptional exposure of virgin flies to vapored nicotine—a major component of E-cigarettes—results in reduced size and weight of offspring across all developmental stages [88]. Moreover, the tracheal length of G1 offspring was reduced, linking the exposure to a feature of the airways. Remarkably, this phenotype was maintained beyond pupation, during which the larval tissue dissolves except for primordia and stem cell populations, from which the adult insect organs are newly formed. This indicates a profound influence of preconception e-nicotine on the G1 generation and cannot be explained by direct toxicity.

Another recent study treated 4-week-old males with cigarette smoke condensate before mating with female mice in an attempt to explain environmental contributions to adolescent diabetes. The study revealed that the body weight of offspring was significantly lower from postnatal weeks 1–6, and the serum glucose levels increased at certain time points in the 8th week of postnatal life [89]. In another study of “adolescent” exposure in mice, male and female mice were exposed to cigarette smoke from the onset of puberty for 6 weeks and mated with room air-exposed control mice [90] . Preconceptional cigarette smoke exposure altered miRNA expression patterns in the spermatozoa compared to room air controls. In G1 progeny from cigarette smoke-exposed fathers, there was a suggested increase in the mean body weight in early postnatal life.

### Mechanisms behind transmission of exposure effects from parents to offspring

Several epigenetic mechanisms are described that may transmit exposure effects from parents to offspring [20–22, 46, 68, 91–94]. For example, in rat, the work of Skinner has shown that a transient exposure of G0 generation gestating females to the agricultural fungicide vinclozolin [95], as well as a range of other toxicant exposures, results in induction of pathology in testis, prostate, kidney, and ovaries of grand offspring, as well as obesity (reviewed in [68]). Such transient toxicant exposure leads to altered differential DNA methylation regions (DMRs) in the G3 generation great-grand offspring [84] and altered ncRNA expression in spermatozoa of male line descendants [96]. The altered ncRNAs in sperm of G1 males appear to be co-located with DMRs observed in subsequent generations [97], suggesting a potential role for these ncRNAs in directing methylation in the zygote postfertilization. Such RNA-directed DNA methylation has previously been demonstrated in plants [98]. There is also evidence that DNA methylation (and/or DNA methyltransferases, etc.) plays a role in paternal influences, regardless of ncRNA patterns [68, 92, 99–103].

Mammalian spermatogenesis is accompanied by profound changes in several classes of small RNAs [104]. MicroRNAs (miRNAs), in particular, are influenced by environmental stimuli and transport epigenetic information via sperm cells to the oocyte. By regulation of gene expressions in the early zygote, miRNAs transmit paternally acquired phenotypes. Evidence for causality of sperm small RNA for transmission of paternal phenotype in mice has been established via injection of sperm RNAs from males exposed to various environmental stimuli into zygotes from unexposed parents, resulting in offspring phenotypes that fully or partially recapitulated the paternal environmental input, including behavioral changes, obesity, and altered glucose metabolism [46, 91, 96, 105, 106].

For example, chronic stress induction in pubertal male mice led to the upregulation of nine miRNAs in their sperm. Injection of these miRNAs into an early zygote recapitulated a blunted stress response in offspring after brief restraint stress, thus demonstrating a direct role of sperm miRNAs in transgenerational inheritance [107]. We speculate that the spermatozoal miRNAs regulate epigenetic changes that are transmittable to offspring, thereby influencing their body weights. These results provide initial experimental evidence that pubertal cigarette smoke exposure may affect infant development.

In addition to altered miRNAs expression, cigarette smoke is reported to cause DNA damage, chromosomal alterations, mutations, and altered DNA methylation in sperm [108–112].

Each of these processes could also potentially influence the phenotype of offspring. The direct link between preconceptional cigarette smoke exposure and molecular signaling and changes in offspring body weight in early life needs further clarification. Hammer et al. identified molecular pathways that could be explored more deeply in this regard, namely, the Hippo and Estrogen signaling pathways [90]. To a large extent, miR-204-5p and miR-96-5p were implicated in the regulation of these developmental pathways. MiRNAs play an important role in regulating lung development [113]; thus, these findings are in agreement with the human studies showing that paternal smoking was associated with more asthma and lower lung function in offspring [12–14]. The functional role of miRNAs in early embryonic development of prospective offspring from smoke-exposed male mice may bring this understanding forward. For example, microinjection of miRNA mimics and inhibitors into the cytoplasm of early zygotes could contribute to determine causality for the changes in sperm miRNAs observed in response to paternal preconceptional smoke exposure in the study of Hammer et al. [90].

While there is an increasing range of exposures that have been shown to lead to transgenerational effects in humans (reviewed in [114]), evidence for epigenetic factors playing a role in these effects in humans is extremely limited, due to the acknowledged difficulties of studying such processes in humans [11]. None the less, some evidence in humans does exist. For example, paternal toxicant exposures and even endurance training have been shown to result in altered ncRNA content of spermatozoa and methylation [110, 115–119]. Involvement of epigenetic mechanisms transferred from father to offspring was suggested by Knudsen et al. based on a preliminary EWAS analyses of 195 Bergen RHINESSA and ECRHS participants, showing that father’s smoking was associated with specific DNA methylation patterns in adult offspring [120]. However, further research is clearly needed in human studies to establish the precise role for epigenetic processes in mediating transgenerational effects in humans.

## Discussion

While the science on human epigenetic inheritance is in its infancy, and even more so when specifically considering inheritance of environmental effects induced around the time of prepuberty, there is already convincing evidence that the environment and exposures in prepuberty may be important for the phenotype of future offspring, in particular for the male line. Analyses of the RHINESSA study consistently demonstrate poorer respiratory health outcomes in offspring of fathers who started tobacco smoking or became overweight in prepuberty, supporting that prepuberty in males may be an important time window with regard to offspring’s respiratory health. Historic studies indicate that ancestral nutrition during prepuberty may influence grand-offspring’s health. The possibility of coming to conclusions on causality based on human data is greatly enhanced by state-of-the-art statistical methods that optimize the gain while accounting for the many complexities and deficiencies in human multigeneration data. Important analyses from the ALSPAC and RHINESSA cohorts show a role of smoking starting in prepuberty for offspring’s excessive fat mass. This finding is supported by a mouse study, suggesting changes in offspring’s weight induced by exposing adolescent male mice to smoking *versus* room air. This latter study also revealed alterations in small RNAs in the spermatozoa of the exposed fathers. A role of sperm small RNA in the transmission of paternal exposures to offspring’s phenotypes has been reasonably well established, and chemical exposures as well as overweight have been shown to influence epigenetic programming in germ cells. Finally, it seems biologically plausible that male prepuberty could be a time window of particular susceptibility, given the extensive epigenetic reprogramming taking place in the spermatocyte precursors at this age. It also seems plausible that the marked sex differences in germ cell maturation could underlie the sex-specific patterns repeatedly observed in human studies. Thus, consistency in results from human studies of various designs, various exposures and outcomes, and different analytical approaches, as well as mechanistic research and biological plausibility, all support the notion that exposures in the prepuberty of males may influence the phenotype of future offspring.

It is intriguing that historic studies suggest grandparental supposedly harmful exposures in prepuberty give *better* health in grand-offspring, while cohort studies find parental harmful exposure in prepuberty gives *poorer* health in offspring. We find that paternal grandmother’s smoking was related to higher asthma risk in grand-offspring but appeared to have a beneficial effect in eliminating the harmful effects of father’s smoking in prepuberty on offspring’s asthma—which was quite substantial if the grandmother did *not* smoke. One might speculate that grandparent exposure, maybe as part of a wide array of effects, somehow also could induce an adaptive response or protective mechanisms.

Puberty starts somewhat earlier in girls than in boys, and Bygren et al. defined the slow growth period thereafter. However, Golding et al. [82] found that higher offspring’s fat mass was related to smoking in parental prepuberty, with *later* onset in girls (11–15 years) than boys (<11 years). There are marked differences in germ cell maturation and development between the sexes, but the details are not fully known and the knowledge on human germ cell development in the key interval between fetal life and puberty when mature sperm and eggs can be studied is limited.

### Moving toward causal inference from imperfect human data: statistical mediation modeling in multigeneration observational studies

Identifying preconception susceptibility windows and investigating mechanisms for exposure effects across generations implicates different methodological challenges. Twenty-first century opportunities to integrate molecular research with epidemiological data increase the possibilities but also the complexity [121]. Real world data on humans are imperfect and researchers often exclude information in order to adapt data to the analytical possibilities. [121] Methods of causal inference [122] can greatly increase the information that can be obtained from observational studies, in the many types of research questions in which studies with randomized exposure cannot be performed. In particular, counterfactual-based mediation models [123] within a hierarchical framework are developed to take the different sources of complexity into account in the analyses:

(i) The variables of interest [exposures, outcomes, mediators (i.e., intermediary variables between exposures and outcomes)] as measured in different life periods (pregnancy, prepuberty, adulthood) and generations (grandparents, parents, offspring)
(ii) The variables of interest as measured at individual or group levels (e.g., data structure in RHINESSA is hierarchical because offspring siblings have the same biological parents and parents are sampled from different centers)
(iii) The causal pathways as investigated among multiple exposures, multiple mediators, and multiple outcomes (MEMMMO framework) [124]

Mediation modeling allows decomposing the total effect of each exposure on each outcome into its natural direct effect (i.e., the effect of the exposure on the outcome via pathways that do not involve the mediators) and its natural indirect (mediated) effect (i.e., the effect of the exposure on the outcome due to the effect of the exposure on the mediators) [125]. The natural (counterfactual-based) direct effect is the change in the expected value of the outcome for the change in exposure status, keeping the mediator at its expected value when the exposure is absent. The natural (counterfactual-based) indirect effect is the change in the expected value of the outcome when the exposure is present, but the mediator changes from its expected value when the exposure is absent to its expected value when the exposure is present.

Measured and unmeasured potential confounders in different generations must be investigated in evaluating causal relationships. In particular, researchers must verify whether the measured potential confounders represent the smallest set of covariates that needs to be included in the models in order to eliminate measured confounding, by using directed acyclic graphs (DAGs) [126]. Potential confounding by some unmeasured variable [127] can be explored by performing probabilistic (Monte Carlo) simulations aimed at assessing whether the estimated effects change after inclusion of the unmeasured confounders in the models [128]. Researchers can also quantify which is the minimum strength of association that an unmeasured confounder would need to have with both the mediator and outcome, conditional on the measured confounders, to fully explain away the observed direct or indirect effects (mediational *E*-values) [129].

In summary, recent advances in statistical mediation modeling and in the analyses of unmeasured confounding can enable researchers to assess whether prepubertal exposures in parents have different direct or indirect effects on offspring’s health, as compared with exposures in other time windows or in grandparents.

### Evidence of causation discussed according to Bradford Hill and Lederer’s principles

Research on parental prepuberty exposures as related to offspring health is limited, but in the following paragraphs, we will discuss the evidence according to a 21st century perspective of the classic criteria by Bradford Hill [121, 130], and the key principles for causal inference in analysis of observational data from Lederer et al. [122]

#### Bradford Hill principles

##### Consistency

There are consistent findings of associations between parental (ancestral) exposures during the prepuberty period with phenotypes in future offspring, most consistent in the male line. This is found for several different exposures in prepuberty, with most evidence on smoking onset, onset of overweight, and living through times of poor harvests. Consistent associations are also found for several phenotypic outcomes in offspring, with most evidence on asthma, lung function, fat mass, and longevity. Further, the findings are consistent between different types of statistical analyses of the data, from simple analyses to advanced statistical approaches, with different ways of accounting for dependency between family members, unmeasured confounding, etc. There are consistent results from analyses of different data sources such as the RHINESSA multigeneration study built on a longitudinal parental cohort, the ALSPAC study built around a birth cohort, and a Guatemalan intervention study; also, three-generation analyses using data from historic studies from Överkalix, Uppsala, and Germany consistently support the notion of a preconception susceptibility window. Finally, epidemiological studies of humans and experimental animal studies show consistent results.

##### Specificity

Several of the studies show effects that are specific for the prepuberty time window, as opposed to exposures occurring after that time window. Sex-specific patterns are typical in this research.

##### Temporality

In this literature, the temporality is always clear: the exposure occurred in ancestors’ prepuberty, before the outcomes in offspring.

##### Biological gradient, dose–response

This could imply, for instance, the number of cigarettes smoked daily in prepubertal boys, a degree of detail that is usually not available in these studies.

##### Biological plausibility

Given knowledge on germ cell development and sex differences in this, as well as findings from experimental mouse models that support epidemiological findings, it is plausible that germ cells may be more vulnerable to exposures in prepuberty following sex-specific patterns.

##### Experimental evidence

The mouse study by Hammer et al. provide evidence that exposure to male mice from adolescence impacts on offspring phenotype, as well as mechanisms by which such exposure may impact offspring. There is further experimental evidence for transfer of exposure effects at any time before conception to offspring phenotype through sperm.

#### Lederer’s key principles

In summary, these state that (i) causal inference requires careful consideration of confounding, by (a) historical confounder definition with purposeful variable selection or (b) by causal models using directed acyclic graphs; (ii) interpretation of results should not rely on the magnitude of *P*-values; and (iii) results should be presented in a granular and transparent fashion [122]. The human studies cited in this review in general follow these principles reasonably well, most of the studies accounting for potential confounding factors by the first method. Recent RHINESSA analyses [13, 76] use increasingly advanced causal statistical models, selecting variables using directed acyclic graphs and accounting for unmeasured confounding; the methodology used in these analyses increases the possibility to approach an interpretation on causality.

*In conclusion*, regarding Bradford Hill’s principles for causation [121, 130], the literature shows consistency and specificity, the temporality is clear, and the results are biologically plausible and supported by experimental evidence. Regarding Lederer’s key principles for causal inference based on observational data, key publications use optimal methodological approaches [13, 14, 76, 77]—and give results consistent with those using simpler analytical methods [12]. We conclude that although this research field is new and the literature limited to relatively few studies, the findings to date give reasonable support to the notion that parental prepuberty exposures might causally affect offspring’s phenotype.

### Challenges and research needs

Addressing the prepubertal time window in parents represents a major challenge, in both human and mechanistic studies. The human reproductive cycle spans decades and the interest in the male line and exposures during prepuberty is of recent date. Thus, there are scarce data on exposures during this time window in cohorts with outcome data on offspring. In animal models, the puberty time window adds difficulty to multigenerational models, because it is so short. In humans the key cell—the germline precursor cell that is maturing during prepuberty—is mostly out of reach for science. Fortunately, such cells can be retrieved from mice. Thus, innovative thinking and multidisciplinary approaches, as well as optimal use of existing human data sources, are of key importance.

#### Human studies

The research question requires quality data on parental exposures during prepuberty, offspring’s outcomes, and potential confounding variables, for a sufficient number of study participants of both generations. Only a few cohorts have such data, but the opportunities will increase with time as the participants of several large birth cohorts are entering the childbearing age. On the other hand, one-generation cohorts with valuable exposure data may have parent-reported data on offspring. These studies usually have limited data on potential confounding variables in offspring and need analyses validating parental reporting of information about their offspring. One-generation studies in which participants provide information about their parents may be less useful in this context, due to the added challenge of reporting parents’ exposures during the prepuberty age. Analyses of the RHINESSA/RHINE/ECRHS studies include validation of asthma as reported by parents and offspring [131], reporting of parental smoking [132], maternal reporting of pregnancy and birth characteristics [133], and retrospective recall of body shapes [134]. It is important that the research teams of multigeneration cohorts give priority to validation studies; this will increase the knowledge that can be obtained from existing human data. There may also be opportunities to follow-up cohorts of children that have undergone interventions, such as the Guatemala study cited above, with regard to subsequent offspring.

#### Registry data

North European countries have registries with high data quality and excellent nationwide coverage. Population and tax registries allow unbiased identification of family members and may contain data on income, occupation and socioeconomic factors. Useful outcome data can be obtained from prescription registries, cause of death registries, etc. Armed forces registries have information of several dimensions on young men at age 18 years; in Norway, this registry dates back to 1876. Kuiper et al. [78] used registry data to generate parental air pollution data with high time resolution from geocoding parental addresses back in time. However, the registries only rarely allow characterization of parental exposures during prepuberty but can add importantly on other aspects.

#### Statistical analyses

Multigeneration data are often highly complex, with the need to account for dependence between family members, multiple exposure windows, multiple mediators, multiple outcomes, sex-specific patterns, and unmeasured potential confounding. Statistical techniques and available software for causal analyses of multigeneration observational studies are rapidly evolving and allow for optimal knowledge from valuable data.

#### Animal models

Few animal models can address prepuberty; for instance, puberty in most strains of mice lasts only a couple of days. However, animal models allow control of the exposure, exploration of germline cells, as well as investigation of offspring and multiple generations. Thus, animal models are needed to establish experimental evidence of preconception exposure effects and the underlying biological mechanisms.

#### Mechanistic research based on human samples

Studies of epigenetic characteristics and gene expression can give important insight into biological mechanisms and functionality. Mechanistic research in humans is needed, for instance; it would be important to attempt to explore, e.g., a potential role of ncRNAs in multigenerational effects through the male germline. However, the study of humans poses limitations as opposed to animal studies that can explore primordial germ cells in relation to parents’ exposures and characteristics in future offspring. In humans, biomaterial from offspring may be available from persons with parental exposure information, and it may be possible to obtain sperm samples from fathers of offspring with relevant data/biomaterial, while it would be ideal to have sperm collected back in time before conception of the offspring. Mechanistic studies in humans thus need to combine several approaches and pieces of information.

#### Behavioral sciences and health economy

The identification of prepuberty as a critical period for the health of both the young and their future offspring implies a need for intensified research efforts on how to reach young boys/girls and induce beneficial behaviors and choices. Further, while the costs and effects of interventions of altered public health strategies usually are estimated with regard to one generation, there is a need for health economic research estimating the potential benefit for two generations, of the target group as well as future offspring.

## Conclusions and public health implications

The evidence presented in this review from epidemiological studies in humans, state-of-the-art causal statistical analyses, experimental mice models, and mechanistic research, all support the notion that exposures in the prepuberty of males may influence the phenotype of future offspring.

Innovative thinking and multidisciplinary approaches, as well as optimal use of existing human data sources, are of key importance for further scientific development. Addressing the parental prepuberty time window is particularly challenging, in human studies in which there is sparse data on this time window in cohorts with offspring data, and in mice models in which puberty may last 2 days. There is a need for more research on every aspect of this topic, from scientists covering a variety of disciplines.

This review gives reasonable evidence that the environment, lifestyle, and behaviors in the prepuberty period may be important for two generations. Hence, by targeting boys and girls early in this age window, returns are likely over the lifespan of the index persons as well as from their future offspring—a double benefit from the invested resources. This time window is feasible to target since both girls and boys aged 10–12 years almost universally go to school, which gives a possibility to reach also disadvantaged children. Also, this age group may be more open-minded to interventions. Early onset of smoking is one factor that according to the reviewed literature may harm future offspring as well as the smoking child. Based on experimental studies showing multigenerational effects of nicotine, it seems likely that also E-cigarettes and oral moist tobacco may pose a double threat to public health. Other mixed chemical exposures in prepubertal children should also be considered with regard to potential impact across generations. Further, there is reasonably convincing evidence that overweight that starts prior to puberty may be harmful to future offspring’s health. Childhood obesity is increasing globally, and a scenario of harmful effects also for the next generation is realistic.

Can multigeneration science open new perspectives on how to improve health, reach the disadvantaged, and gain more health from limited resources? We believe, yes, that research on identifying susceptible time windows that are important for the health of the target person as well as future offspring may open new opportunities for efficient and sustainable public health strategies aimed at improving health and reducing social inequalities.

The evidence presented in this review opens for radical rethinking of preventive strategies. Scientists need to make policy makers and health strategy planners aware of the concept that *improvements* to the health and conditions of school children are likely to benefit not only the children themselves but also their future offspring. However, on the other hand, *deterioration* in the living conditions of schoolchildren, such as those millions of children are experiencing now during the current pandemic, may have adverse consequences also for the next generation. Extensive and repeated dissemination of these concepts and new results to stakeholders in public health is of key importance.

In conclusion, the identification of a susceptibility window in prepuberty that is important for both children and their future offspring can lead to a paradigm in public health policies in which strategically targeted interventions in prepuberty children may benefit two generations at the same time. It is crucial to use this new knowledge to develop efficient and sustainable systems to prevent diseases and improve health.

## Data Availability

There are no new data associated with this article. No new data were generated or analyzed in support of this research.

## Notes

**†Grant Support:** Cecilie Svanes was supported by a grant from the Escher Family Fund/Silicon Valley Community Foundation. Co-ordination of the **RHINESSA** study has received funding from the Research Council of Norway (Grants No. 274767, 214123, 228174, 230827, and 273838), ERC StG project BRuSH #804199, the European Union's Horizon 2020 research and innovation programme under grant agreement No. 633212 (the ALEC Study WP2), the Bergen Medical Research Foundation, and the Western Norwegian Regional Health Authorities (Grants No. 912011, 911892, and 911631). Study centers have further received local funding from the following: Bergen: the above grants for study establishment and co-ordination, and, in addition, World University Network (REF and Sustainability grants), Norwegian Labour Inspection and the Norwegian Asthma and Allergy Association. Albacete and Huelva: Sociedad Española de Patología Respiratoria (SEPAR) Fondo de Investigación Sanitaria (FIS PS09). Gøteborg, Umeå and Uppsala: the Swedish Heart and Lung Foundation, the Swedish Asthma and Allergy Association. Reykjavik: Iceland University. Melbourne: NHMRC Project Grant ID 1128450, Melbourne University. Tartu: the Estonian Research Council (Grant No. PUT562). Århus: The Danish Wood Foundation (Grant No. 444508795), the Danish Working Environment Authority (Grant No. 20150067134), Aarhus University (PhD scholarship). The **RHINE** study received funding by Norwegian Research Council, Norwegian Asthma and Allergy Association, Danish Lung Association, Swedish Heart and Lung Foundation, Vårdal Foundation for Health Care Science and Allergy Research, Swedish Asthma and Allergy Association, Swedish Lung Foundation, Icelandic Research Council, and Estonian Science Foundation. The co-ordination of **ECRHS** was supported by the European Union's Horizon 2020 research and innovation programme under grant agreement No. 633212 (the ALEC study), the Medical Research Council (ECRHS III), and the European Commission FP5 and FP7 (ECRHS I and II). ECRHS IV is being planned and the 10 RHINESSA study centers are funded by ERC StG project BRuSH #804199 and by the European Union’s Horizon 2020 research and innovation programme under grant agreement No. 874703 (the EPHOR project). The funders were not involved in the study design or in the collection, analysis, or interpretation of the data. This publication is the work of the authors and they will serve as guarantors for the contents of this paper.
